# Unconventional and Powerful Ion Sources for Solid-State Ion Exchange, Cu_2_SO_4_ and Cu_3_PO_4_: Exemplified by the Synthesis of Metastable β-CuGaO_2_ from Stable β-LiGaO_2_

**DOI:** 10.1021/acs.inorgchem.4c05078

**Published:** 2025-01-27

**Authors:** Issei Suzuki, Kako Washizu, Daiki Motai, Masao Kita, Takahisa Omata

**Affiliations:** † Institute of Multidisciplinary Research for Advanced Materials, Tohoku University, Sendai, Miyagi 980-8577, Japan; ‡ Department of Mechanical Engineering, National Institute of Technology, Toyama College, Toyama 939-8630, Japan

## Abstract

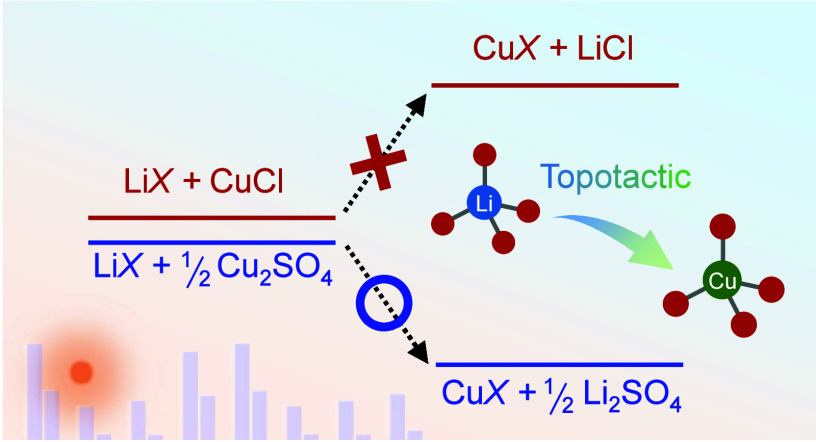

This study introduces a new method for synthesizing Cu^+^-containing metastable phases through ion exchange. Traditionally, CuCl has been used as a Cu^+^ ion source for solid-state ion exchanges; however, its thermodynamic driving force is often insufficient for complete ion exchange with Li^+^-containing precursors. First-principles calculations have identified Cu_2_SO_4_ and Cu_3_PO_4_ as more powerful alternatives, providing a higher driving force than CuCl. It has been experimentally demonstrated that these ion sources can open up new reaction pathways through experimental ion exchanges, such as from β-LiGaO_2_ to β-CuGaO_2_, which were previously unattainable. An important perspective provided by this study is that the potential of such simple compounds to act as powerful ion sources has been overlooked and that they were identified through straightforward first-principles calculations. This work presents the initial strategic design of an ion-exchange reaction by exploring suitable ion sources, thereby expanding the potential for synthesizing metastable materials.

Solid-state ion exchange is an efficient method for synthesizing metastable compounds. This technique entails topotactically substituting ions in a precursor that has the same or a similar crystal structure as the target materials while preserving the crystal framework, as illustrated by reaction 1.^1−4^
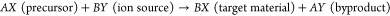
1

Solid-state ion exchange generally occurs at relatively low temperatures (150–600 °C), preventing structural reconstruction into the most thermodynamically stable phase and enabling access to metastable phases not found on phase diagrams. In ion exchange, four chemical species are involved: the precursor and ion source (reactant system), as well as the target material and byproduct (product system). The reaction is driven by the overall change in the Gibbs free energy (Δ_r_*G* = Δ_r_*H* – *T*Δ_r_*S*).^4^ A recent study has shown that the enthalpy change (Δ_r_*H*) of ion-exchange reactions, evaluated by first-principles calculations, can be used to screen whether the reaction will proceed because the entropic gain due to ion mixing (−*T*Δ_r_*S*) in relatively low temperatures is negligible when Δ_r_*H* is several tens of kilojoules per mole.^5^ A crucial yet previously underappreciated aspect of ion exchange is that, even when the target material is fixed, Δ_r_*G* of the overall reaction (i.e., whether the reaction will proceed or not) can be controlled by altering the combination of the reactant system. Conventional studies on ion exchanges, except for those involving Ag^+^ ion exchange with AgNO_3_, have predominantly utilized chloride salts such as CuCl or CoCl_2_ as ion sources^3,6−9^ with no investigation into alternative ion sources. Consequently, if chloride salts failed to react with a particular precursor, further attempts to establish that reaction pathway were discontinued.^10^ By strategically designing a new equilibrium field governed by four chemical species and exploring novel ion sources, researchers can significantly expand the accessible range of metastable materials.

In this study, we investigate ion exchange from Li^+^-containing precursors to Cu^+^-containing oxides as an illustrative example to explore new powerful ion sources through first-principles calculations. Additionally, we demonstrate that novel reaction pathways can be developed experimentally by utilizing such ion sources.

In the synthesis of Cu^+^-containing oxides through ion exchange, Na^+^-containing precursors have primarily been utilized,^6,7,11,12^ while Li^+^-containing precursors have been restricted to certain layered compounds.^8,13^ This limitation arises from the greater stability of Li^+^-containing oxides compared to Na^+^-containing oxides, coupled with the insufficient driving force for ion exchange provided by the CuCl ion source.^5^ This contrast is evident in the ion-exchange process for β-CuGaO_2_: reaction 2 involving a β-NaGaO_2_ precursor, which exhibits a negative calculated Δ_r_*H*, results in complete ion exchange to yield β-CuGaO_2_. Conversely, reaction 3 involving a β-LiGaO_2_ precursor with a positive Δ_r_*H* does not lead to successful ion exchange experimentally.^5^
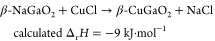
2
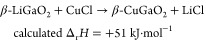
3

The ionic radius of Na^+^ (1.00 Å for 4-fold coordination) is significantly larger than those of Cu^+^ and Li^+^ (0.60 and 0.59 Å, respectively), leading to general challenges in ion exchanges from Na^+^ to Cu^+^, such as phase transition induced by coordination number changes and severe cracking due to volume shrinkage.^14,15^ Additionally, Na^+^-containing precursors often suffer from Na deficiency due to the high vapor pressure of Na,^16−18^ resulting in severe cation deficiency in the obtained target materials (see a detailed explanation in section S1).^14^ These challenges can be addressed using Li^+^-containing precursors.

To identify powerful ion sources with ample driving force for ion exchange from Li^+^ to Cu^+^, we calculated the enthalpy difference between the Cu^+^-containing salts and their Li^+^-containing counterparts. Using the identified Cu^+^ ion source, an ion-exchange pathway from β-LiGaO_2_ to β-CuGaO_2_ was demonstrated. This demonstration validates that investigating ion sources can unveil previously inaccessible ion-exchange pathways.

The formation enthalpies of Cu^+^-containing salts (CuCl, CuBr, CuI, Cu_2_SO_4_, Cu_3_PO_3_, CuCN, CuSCN, and CuH) and their Li^+^-containing counterparts at 0 K were evaluated through first-principles calculations. Detailed calculation conditions and initial structures are provided in section S2. Ion exchange was experimentally conducted by mixing a Li^+^-containing precursor with the ion source in a Cu:Li = 1:1 ratio, followed by heating under vacuum (see details of synthesis precursors, the preparation of commercially unavailable Cu_2_SO_4_^19^ and Cu_3_PO_4_,^20^ and the ion-exchange process in sections S3 and S4). The reaction products were identified using X-ray diffraction (XRD; SmartLab, Rigaku, Japan), and the lattice parameters and relative proportions of impurity phases were determined through Rietveld analysis (see details of Rietveld analysis in section S5). The chemical compositions were determined by dissolving the powder samples in a nitric acid solution in an autoclave, followed by inductively coupled plasma (ICP) analysis (Optima 3300XL, PerkinElmer, US).

Figure 1a presents the computed formation enthalpies of various Cu^+^-containing salts and their corresponding Li^+^-containing counterparts. Among the halide salts, CuCl exhibited the most negative Δ_r_*H* for ion exchange, possibly elucidating why CuCl has traditionally been the preferred ion source in earlier ion-exchange studies given its easy availability. In contrast, using Cu_2_SO_4_ and Cu_3_PO_4_ as ion sources yielded even more negative Δ_r_*H* values. Specifically, the calculated driving forces of these salts were higher by 81 and 58 kJ·mol^–1^, respectively, than those of CuCl.

**Figure 1:**
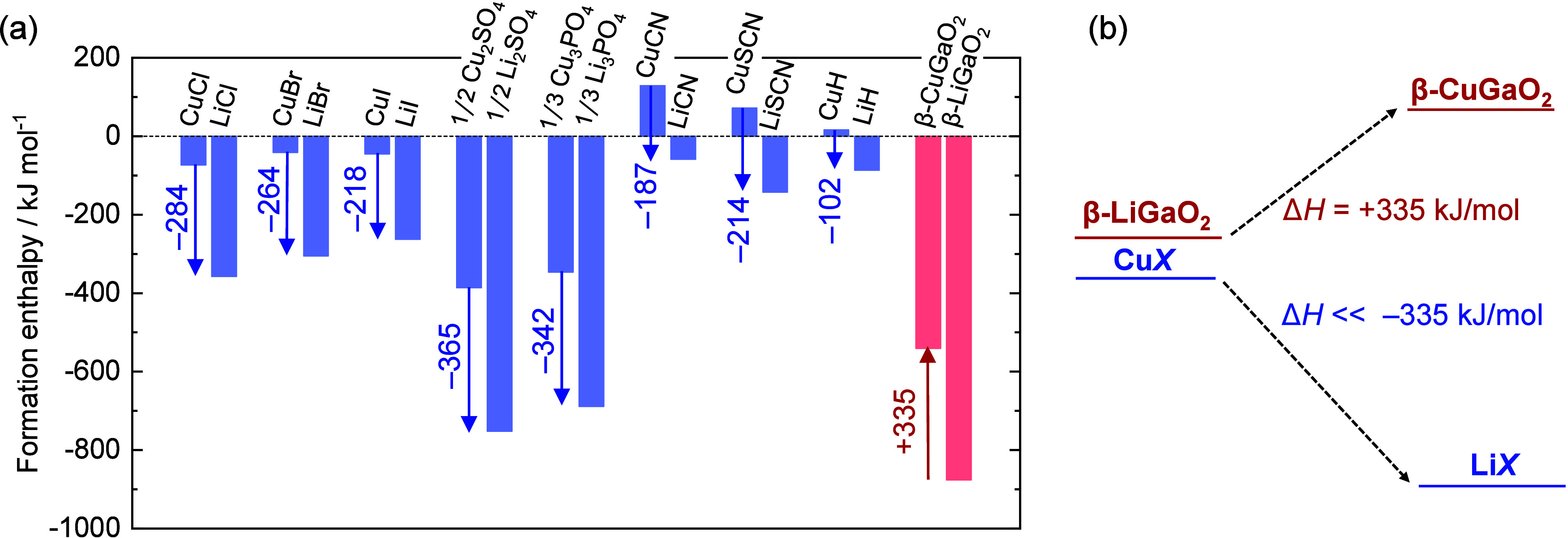
(a) Formation enthalpies of Cu^+^-containing compounds and their Li^+^-containing counterparts. (b) Schematic energy diagram of ion exchange between β-LiGaO_2_ and a Cu^+^-containing ion source.

To demonstrate that these ion sources are more powerful than CuCl, the synthesis of β-CuGaO_2_ from β-LiGaO_2_ described above should be a good case. Because the Δ_r_*H* of β-LiGaO_2_ and β-CuGaO_2_ is +335 kJ·mol^–1^, an ion source and byproduct combination with a Δ_r_*H* more negative than −335 kJ·mol^–1^ is expected to drive the ion exchange (Figure 1b). The overall Δ_r_*H* value for ion exchange using either Cu_2_SO_4_ or Cu_3_PO_4_ as the ion source (reactions 4 and 5) is −30.2 or −6.8 kJ·mol^–1^, respectively, indicating potential reaction progression. However, this expectation is solely based on the thermodynamic perspective at 0 K. To complete ion exchange within a reasonable time frame at experimental temperature, sufficiently high interdiffusion coefficients of Cu^+^ and Li^+^ in these ion sources are necessary.^21^ This was investigated using the following ion-exchange experiment.
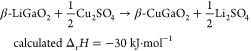
4
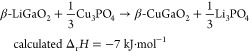
5

Parts b and c of Figure 2 show the XRD profiles of β-LiGaO_2_ after heating with either CuCl or Cu_2_SO_4_. When CuCl was used as the ion source, β-LiGaO_2_ remained unchanged, indicating no ion exchange, in line with a previous report.^5^ Conversely, using Cu_2_SO_4_ as an ion source resulted in the formation of β-CuGaO_2_ and the byproduct Li_2_SO_4_, indicating successful ion exchange. Also, the product exhibited black color reflecting a β-CuGaO_2_ band gap of 1.5 eV^6^ (inset in Figure 2c). The byproduct was eliminated by water washing. The Cu_2_O impurity likely originated from partial decomposition of the Cu_2_SO_4_ ion source. During Cu^+^ ion exchange, trace impurities such as Cu_2_O and metallic Cu are commonly produced. These impurities can typically be eliminated by washing with aqueous ammonia, facilitating isolation of the single-phase target material.^8^ However, this method is unsuitable for β-CuGaO_2_ due to its solubility in aqueous ammonia because of the amphoteric nature of Ga. The chemical composition of the water-washed sample, as determined through ICP analysis, was Li:Cu:Ga:S = 0.014:1.23:1:0.026. The higher Cu content compared to that of Ga is attributed to the presence of the Cu_2_O impurity phase. Rietveld analysis determined the molar ratio of β-CuGaO_2_:Cu_2_O = 86.8:13.2 corresponding to an atomic ratio of Cu:Ga = 1.30:1, which is consistent with ICP analysis. The low Li content suggests almost complete replacement of Li^+^ with Cu^+^. Furthermore, the lattice constants of the resulting β-CuGaO_2_ (*a*_0_ = 5.472 Å, *b*_0_ = 6.609 Å, and *c*_0_ = 5.262 Å) closely matched the reported values (*a*_0_ = 5.460 Å, *b*_0_ = 6.610 Å, and *c*_0_ = 5.274 Å),^22^ further confirming nearly complete ion exchange. These results demonstrate that using Cu_2_SO_4_ as the ion source allows access to ion exchange that is unattainable with CuCl.

**Figure 2:**
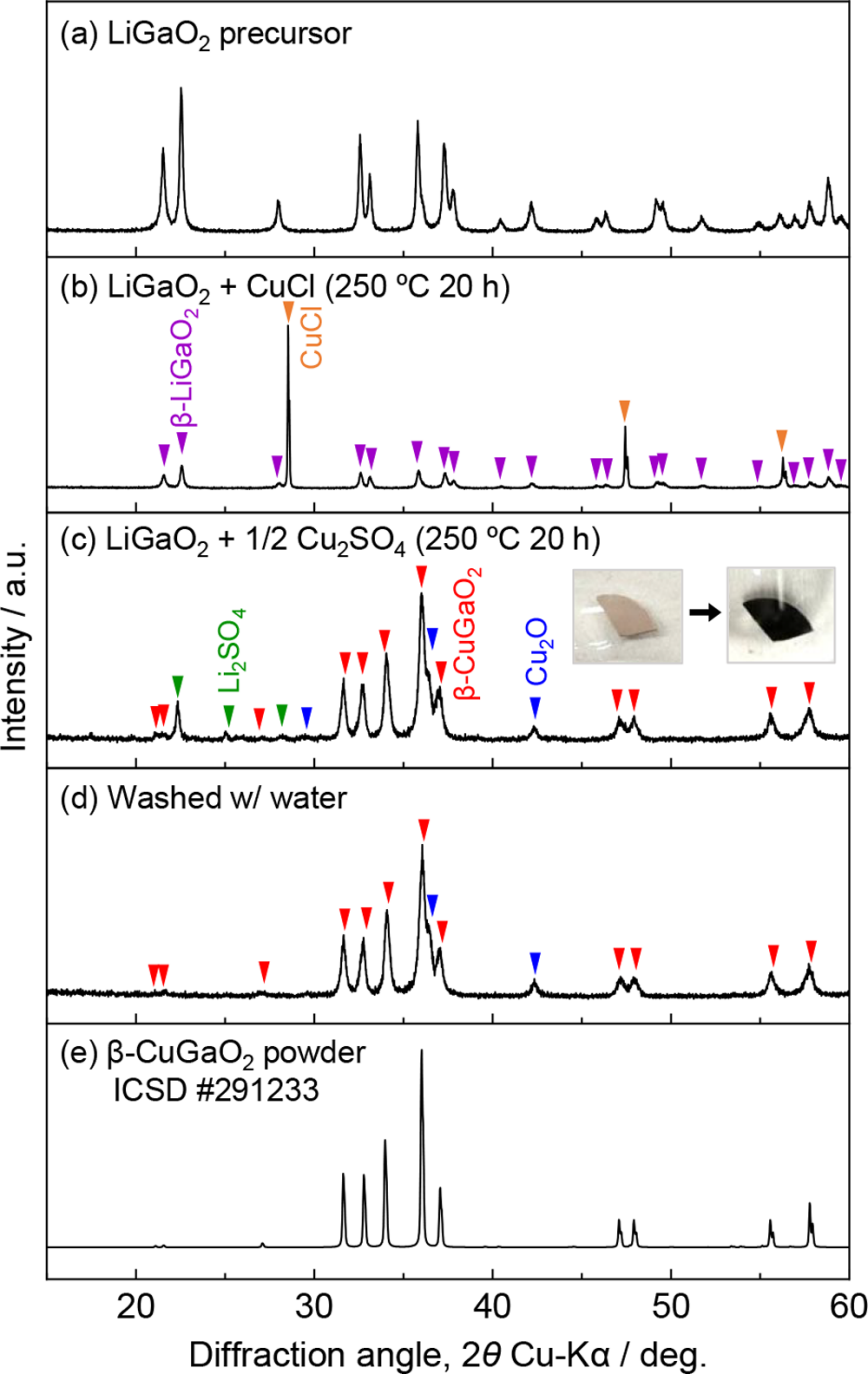
XRD profiles of the samples after the heating process: (b) β-LiGaO_2_ and CuCl without washing, β-LiGaO_2_ and Cu_2_SO_4_ (c) before and (d) after washing along with (a) the profile of the β-LiGaO_2_ precursor and (e) the simulated powder pattern of β-CuGaO_2_ (ICSD 291233^22^). The inset in part c shows photographs of the sample before and after heating.

In the case of Cu_3_PO_4_ as an ion source (reaction 5), in contrast, β-LiGaO_2_ remained unchanged after heating at 250 °C (see section S6). Additionally, the reverse reaction (i.e., the reaction between Li_3_PO_4_ and β-CuGaO_2_) also did not proceed at 250 °C (see section S7). These results indicate that Δ_r_*H* for this reaction is almost zero at the actual experimental temperature, considering the facts that uncertainty in determining the enthalpy of metal oxides by first-principles calculations has a standard deviation of 24 meV·atom^–1^ ^23^ (equivalent to 2.3 kJ·mol^–1^ in this case) and that the calculated Δ_r_*H* is based on 0 K without considering temperature effects. Nevertheless, the complete ion exchange of reaction 6 was experimentally achieved (Figure 3a,b). The direction of this reaction provides direct evidence that Cu_3_PO_4_ functions as a more powerful ion source than CuCl. Cu_2_SO_4_ is prone to instability in air,^24^ while Cu_3_PO_4_ offers enhanced atmospheric stability. Therefore, Cu_3_PO_4_ would be utilized as an easy-to-handle and more powerful Cu^+^ ion source than CuCl, particularly in ion-exchange processes where a driving force as high as that needed for β-LiGaO_2_ is not required.
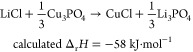
6

**Figure 3:**
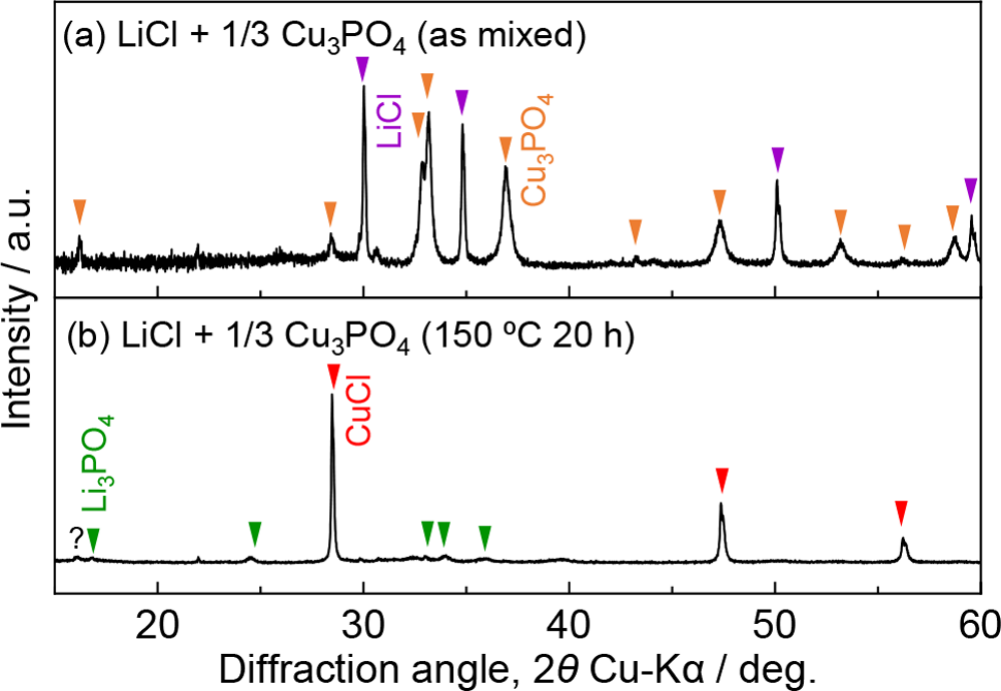
XRD profiles of LiCl and Cu_3_PO_4_ (a) before and (b) after heating.

Cu_2_SO_4_ and Cu_3_PO_4_ undergo partial decomposition at 200–250 °C (Figures 2c and S4). The robust driving forces of Cu_2_SO_4_ and Cu_3_PO_4_ for ion exchange originate from their metastable nature.^19,25^ Nevertheless, these ion sources possess enough thermal stability for ion exchange with β-LiGaO_2_ and LiCl, as demonstrated above, because monovalent cation exchange in oxides typically proceeds at 150–250 °C.^5^ It should be noted here that the balance between the driving force of the ion source and its thermal instability could be more significant for multivalent cation exchange, which often requires higher reaction temperatures due to relatively low interdiffusion coefficients.^4^

In summary, Cu_2_SO_4_ and Cu_3_PO_4_ were identified in this study as ion sources with stronger driving forces for ion exchange with Li^+^-containing precursors compared with the conventional ion source CuCl. The use of Cu_2_SO_4_ facilitates ion exchange from stable β-LiGaO_2_ to metastable β-CuGaO_2_, a reaction pathway previously considered unachievable. While Cu_3_PO_4_ did not provide a sufficient driving force for ion exchange with β-LiGaO_2_, it is a user-friendly and more powerful ion source than CuCl. It is highly intriguing that such simple compounds as Cu_2_SO_4_ and Cu_3_PO_4_ have been overlooked as powerful tools for inducing topotactic reactions and that they were identified through straightforward first-principles calculations. Ion exchange is not limited to monovalent ions in oxides; it is also applicable to multivalent ions and other material groups, such as chalcogenides and pnictides,^25^ implying that many powerful ion sources may remain undiscovered. This study is expected to serve as a starting point for accelerating further exploration of unconventional ion-exchange pathways and expanding the possibilities for synthesizing new inorganic metastable materials.

## Supporting Information Available

Explanation of the difficulties in ion exchange involving Na^+^-containing oxide precursors, calculation conditions, synthesis procedures of β-LiGaO_2_, Cu_2_SO_4_, and Cu_3_PO_4_, Rietveld analysis, and supplementary results of ion exchange (PDF)

## References

[ref1] Gabilondo E.; O’Donnell S.; Newell R.; Broughton R.; Mateus M.; Jones J. L.; Maggard P. A. Renaissance of Topotactic Ion-Exchange for Functional Solids with Close Packed Structures. Chem. Eur. J. 2022, 28, e202200479 10.1002/chem.202200479. 35389540 PMC9321548

[ref2] Parija A.; Waetzig G. R.; Andrews J. L.; Banerjee S. Traversing Energy Landscapes Away from Equilibrium: Strategies for Accessing and Utilizing Metastable Phase Space. J. Phys. Chem. C 2018, 122, 25709–25728. 10.1021/acs.jpcc.8b04622.

[ref3] Haraguchi Y.; Nishio-Hamane D.; Matsuo A.; Kindo K.; Katori H.A. High-temperature magnetic anomaly via suppression of antisite disorder through synthesis route modification in a Kitaev candidate Cu_2_IrO_3_. J. Phys.: Condens. Matter 2024, 36, 405801 10.1088/1361-648X/ad5d3a. 38941989

[ref4] Nakamura T.; Kasai K.; Iura J.-i. Substitution of Ba^2+^ for Ca^2+^ in the solid–liquid system: Ba*M*O_3_(s)–CaCl_2_(l) (*M* = Ti, Zr, Ce). Proceedings of the First International Symposium on Molten Salt Chemistry and Technology, April 20–22, 1983, Kyoto, Japan; Electrochemical Society of Japan, 1983; pp 379–382.

[ref5] Suzuki I.; Kita M.; Omata T. Designing Topotactic Ion-Exchange Reactions in Solid-State Oxides Through First-Principles Calculations. Chem. Mater. 2024, 36, 4196–4203. 10.1021/acs.chemmater.3c03016.

[ref6] Omata T.; Nagatani H.; Suzuki I.; Kita M.; Yanagi H.; Ohashi N. Wurtzite CuGaO_2_: a new direct and narrow band gap oxide semiconductor applicable as a solar cell absorber. J. Am. Chem. Soc. 2014, 136, 3378–3381. 10.1021/ja501614n. 24555768

[ref7] Kita M.; Suzuki I.; Ohashi N.; Omata T. Wurtzite-Derived Quaternary Oxide Semiconductor Cu_2_ZnGeO_4_: Its Structural Characteristics, Optical Properties, and Electronic Structure. Inorg. Chem. 2017, 56, 14277–14283. 10.1021/acs.inorgchem.7b02379. 29083882

[ref8] O’Donnell S.; Kremer R. K.; Maggard P. A. Metastability and Photoelectrochemical Properties of Cu_2_SnO_3_ and Cu_2–*x*_Li_*x*_TiO_3_: Two Cu(I)-Based Oxides with Delafossite Structures. Chem. Mater. 2023, 35, 1404–1416. 10.1021/acs.chemmater.2c03563.

[ref9] Nagatani H.; Mizuno Y.; Suzuki I.; Kita M.; Ohashi N.; Omata T. Variation of crystal structure and optical properties of wurtzite-type oxide semiconductor alloys of β-Cu(Ga,Al)O_2_. J. Appl. Phys. 2017, 121, 235103 10.1063/1.4985700.

[ref10] Dloczik L. CuAlO_2_ prepared by ion exchange from LiAlO_2_. Thin Solid Films 2004, 451–452, 116–119. 10.1016/j.tsf.2003.11.034.

[ref11] Horie H.; Iwase A.; Kudo A. Photocatalytic Properties of Layered Metal Oxides Substituted with Silver by a Molten AgNO_3_ Treatment. ACS Appl. Mater. Interfaces 2015, 7, 14638–14643. 10.1021/acsami.5b01555. 26099451

[ref12] Maruyama Y.; Irie H.; Hashimoto K. Visible light sensitive photocatalyst, delafossite structured α-AgGaO_2_. J. Phys. Chem. B 2006, 110, 23274–23278. 10.1021/jp063406s. 17107176

[ref13] Watanabe K.; Iwashina K.; Iwase A.; Nozawa S.; Adachi S.-i.; Kudo A. New Visible-Light-Driven H_2_- and O_2_-Evolving Photocatalysts Developed by Ag(I) and Cu(I) Ion Exchange of Various Layered and Tunneling Metal Oxides Using Molten Salts Treatments. Chem. Mater. 2020, 32, 10524–10537. 10.1021/acs.chemmater.0c03461.

[ref14] Suzuki I.; Nagatani H.; Kita M.; Omata T. Fabrication of β-CuGaO_2_ thin films by ion-exchange of β-NaGaO_2_ thin films. Appl. Phys. Express 2017, 10, 095501 10.7567/APEX.10.095501.

[ref15] Suzuki I.; Tanemura M.; Omata T. Orientation control of β-NaGaO_2_ thin film: a precursor for β-CuGaO_2_ as a thin-film solar cell absorber. Journal of the Ceramic Society of Japan 2017, 125, 872–875. 10.2109/jcersj2.17157.

[ref16] Ohta H.; Kim S.-W.; Ohta S.; Koumoto K.; Hirano M.; Hosono H. Reactive Solid-Phase Epitaxial Growth of Na_*x*_CoO_2_ (*x* ∼ 0.83) via Lateral Diffusion of Na into a Cobalt Oxide Epitaxial Layer. Cryst. Growth Des. 2005, 5, 25–28. 10.1021/cg049818c.

[ref17] Takahashi Y.; Kataoka K.; Ohshima K.-i.; Kijima N.; Awaka J.; Kawaguchi K.; Akimoto J. Single-crystal synthesis, structure analysis, and physical properties of the calcium ferrite-type Na_*x*_Ti_2_O_4_ with 0.558 < *x* < 1. J. Solid State Chem. 2007, 180, 1020–1027. 10.1016/j.jssc.2006.12.023.

[ref18] Suzuki S.; Suzuki I.; Omata T. Pulsed laser deposition of β-NaGaO_2_: significant dependence of sodium fraction, morphology, and phases of the film on deposition position in the plume. Jpn. J. Appl. Phys. 2023, 62, 035502 10.35848/1347-4065/acbbff.

[ref19] Berthold H. J.; Born J.; Wartchow R. The crystal structure of copper(I)sulfate Cu_2_SO_4_. Zeitschrift für Kristallographie - Crystalline Materials 1988, 183, 309–318. 10.1524/zkri.1988.183.14.309.

[ref20] Snyder K.; Raguž B.; Hoffbauer W.; Glaum R.; Ehrenberg H.; Herklotz M. Lithium Copper(I) Orthophosphates Li_3–*x*_Cu_*x*_PO_4_: Synthesis, Crystal Structures, and Electrochemical Properties, Zeitschrift für anorganische und allgemeine. Chemie 2014, 640, 944–951. 10.1002/zaac.201300606.

[ref21] England W. A.; Goodenough J. B.; Wiseman P. J. Ion-exchange reactions of mixed oxides. J. Solid State Chem. 1983, 49, 289–299. 10.1016/S0022-4596(83)80006-1.

[ref22] Nagatani H.; Suzuki I.; Kita M.; Tanaka M.; Katsuya Y.; Sakata O.; Miyoshi S.; Yamaguchi S.; Omata T. Structural and thermal properties of ternary narrow-gap oxide semiconductor; wurtzite-derived β-CuGaO_2_. Inorg. Chem. 2015, 54, 1698–1704. 10.1021/ic502659e. 25651414

[ref23] Hautier G.; Ong S.P.; Jain A.; Moore C.J.; Ceder G. Accuracy of density functional theory in predicting formation energies of ternary oxides from binary oxides and its implication on phase stability. Phys. Rev. B 2012, 85, 155208 10.1103/PhysRevB.85.155208.

[ref24] Gill D. S.; Singh R.; Rana D. S.; Wagler J.; Kroke E. Preparation, Characterization, X-Ray Structure Determination and Solution Properties of some Novel Copper(I) Bisulfate and Sulfate Salts and Their Stable Derivatives. Z. Naturforsch. B 2011, 66, 1042–1048. 10.5560/ZNB.2011.66b1042.

[ref25] Bamberger C. E.; Specht E. D.; Anovitz L. M. Crystalline Copper Phosphates: Synthesis and Thermal Stability. J. Am. Ceram. Soc. 1997, 80, 3133–3138. 10.1111/j.1151-2916.1997.tb03241.x.

