# Supplementary material for: Unconventional and Powerful Ion Sources for Solid-State Ion Exchange, Cu2SO4 and Cu3PO4: Exemplified by the Synthesis of Metastable β-CuGaO2 from Stable β-LiGaO2
Source: Inorg Chem. 2025 Jan 27;64(5):2165–9. doi: 10.1021/acs.inorgchem.4c05078 (PMC11815836; doi:10.1021/acs.inorgchem.4c05078)
Supplement: Supplementary file 1 — ic4c05078_si_001.pdf [file ic4c05078_si_001.pdf]

Supporting information for

Unconventional and Powerful Ion Sources for Solid-State Ion Exchange, Cu_2SO_4 and Cu_3PO_4: Exemplified by Synthesis of Metastable β-$CuGaO_2$ from Stable β-$LiGaO_2$

Issei Suzuki[*,1], Kako Washizu[1], Daiki Motai[1], Masao Kita[2], and Takahisa Omata[1]

1. Institute of Multidisciplinary Research for Advanced Materials, Tohoku University, Sendai, Miyagi 980-8577, Japan

2. Department of Mechanical Engineering, National Institute of Technology, Toyama College, Toyama 939-8630, Japan

*Corresponding author: issei.suzuki@tohoku.ac.jp

Section S1. Challenges associated with ion exchanges involving Na^+-containing precursors

The ionic radius of Na^+ (1.00 Å for four-fold coordination) is notably larger than that of Cu^+ (0.59 Å). This difference poses challenges in ion-exchange reactions that aim to maintain the crystal framework of the Na^+-containing precursor. To address these challenges, it is advisable to utilize the Li^+-containing precursor since the ionic radius of Li^+ (0.60 Å) closely matches that of Cu^+.

(i) Na^+ tends to exhibit six-fold coordination rather than four-fold coordination in oxides due to its large ionic radius, while Cu^+ generally prefers two- or four-fold coordination. This distinction is evident in $LiMn_2O_4$ and $LiTi_2O_4$, which feature spinel-type structures with Li^+ in four-fold coordination, whereas the Na^+-containing counterparts ($NaMn_2O_4$ and $NaTi_2O_4$) adopt $CaFe_2O_4$-type structures with Na^+ in six-fold coordination.[1] Numerous instances in inorganic chemistry demonstrate this phenomenon, such as $LiVO_3$, where some Li^+ ions are four-fold coordinated, while all Na^+ ions in $NaVO_3$ are six-fold coordinated. Substituting six-fold coordinated Na^+ with Cu_+ would induce a change in the coordination environment and potentially lead to a phase transition, impacting the synthesis of the desired material (unless such a transition is intentionally sought).

(ii) Even when maintaining the coordination environment during the ion exchange from Na^+ to Cu^+, the disparity in ionic radii causes significant volume contraction. Reports indicate that transitioning from β-$NaGaO_2$ to β-$CuGaO_2$, whether in thin-film or single-crystalline forms, results in notable sample cracking. The use of a Li^+-containing precursor, with a lattice size typically closer to that of Cu^+-containing target materials, is anticipated to mitigate cracking. For example, Table S1 summarizes the lattice sizes of β-$CuGaO_2$, β-$NaGaO_2$, and β-$LiGaO_2$.

(iii) In addition to challenges related to differences in ionic radii, Na^+-containing oxides also encounter technical obstacles in achieving a stoichiometric composition without Na deficiency, primarily because of the inherently high vapor pressure of Na (e.g., $5×10^5$ Pa at 800 °C[2]).[3-5] Since the total amount of cations before and after ion exchange remain the same, a precursor with a stoichiometric composition is essential to prevent cation deficiency after ion exchange . In contrast, the significantly lower vapor pressure of Li ($2×10^{-1}$ Pa at 800 °C[2]) facilitates the attainment of a stoichiometric composition in Li+-containing precursors.

Table S1. Lattice parameters of β-$CuGaO_2$[6], β-$NaGaO_2$[7] and β-$LiGaO_2$[8], along with the differences.

	β-$CuGaO_2$	β-$NaGaO_2$	β-$LiGaO_2$
a_0	5.4600 Å	5.498 Å (-0.70%)	5.402 Å (-1.06%)
b_0	6.6101 Å	7.206 Å (+9.01%)	6.372 Å (-3.60%)
c_0	5.2742 Å	5.298 Å (+0.45%)	5.007 Å (-5.07%)

Section S2. Calculation conditions

Calculations were performed on β-MGaO$_2$ (M = Li^+, Cu^+), MCl, MBr, MI, M_2SO_4, M_3PO_3, MCN, MSCN, and MH. The total enthalpies of these compounds were determined using first-principles calculations with the open-source Quantum Espresso software (version 5.2),[9, 10] with the Winmostar V11 GUI (X-Ability Co. Ltd., Japan). Formation enthalpies were obtained by calculating the total enthalpies of elemental substances (Cu, Ga, O_2, Cl_2, Br_2, I_2, S, P, C, N_2, and H_2). Projector-augmented wave-type pseudopotentials were utilized, generated with the 'atomic' code developed by Dal Corso (version 5.0.2) using scalar relativistic computations. The energy cutoffs for plane waves and charge density were set at 100 Ry (1.36 keV) and 900 Ry (12.3 keV), respectively. The self-consistent field (SCF) convergence threshold was set to 1×10^{-7} Ry (1.36×10^{-6} eV). Convergence thresholds for total energy, ionic minimization, and pressure in variable cell relaxation were 2×10^{-5} Ry (2.7×10^{-4} eV), 3×10-4 Ry·Bohr^{-1} (1.2×10^{-5} eV·Å^{-1}), and 12.5 MPa, respectively. The initial structures and geometry optimization parameters are provided in Table S2.

Table S2. Initial crystal structures used for geometric optimization in the DFT calculations obtained from either the ICSD database or the Materials Project (MP)[11]. The space group, *k*-point mesh, and calculated total enthalpies (referenced to the pseudopotentials of the respective elements) are also summarized.

Composition	Initial structure	Space group	*k*-point	Total enthalpy / kJ·mol^{-1}
β-$LiGaO_2$	ICSD 18152[8]	Pna2$_1$ (33)	5×4×5	–489100.4552
β-$CuGaO_2$	ICSD 291233[6]	Pna2$_1$ (33)	5×4×5	–745406.1894
LiCl	ICSD 27981 [12]	Fm-3m (225)	4×4×4	–122231.1863
CuCl	ICSD 78270 [13]	F-43m (216)	7×7×7	–378588.0957
LiBr	ICSD 52236 [14]	Fm-3m (225)	4×4×4	–283590.2986
CuBr	ICSD 78274 [13]	F-43m (216)	4×4×4	–539967.6342
LiI	ICSD 414244 [15]	Fm-3m (225)	3×3×3	–509373.7345
CuI	ICSD 30363 [16]	P3m1 (156)	3×3×1	–765797.4729
Li_2SO_4	ICSD 2512 [17]	P12$_1$/c1 (14)	2×3×2	–341222.1799
Li_2SO_4	ICSD 153806 [18]	Cmcm (63)	3×3×2	–341195.9445
Cu_2SO_4	ICSD 40452 [19]	FdddZ (70)	2×2×2	–853773.1019
Li_3PO_4	ICSD 10257 [20]	Pmn2$_1$ (31)	2×3×3	–347395.7212
Li_3PO_4	ICSD 77095 [21]	Pnma (62)	1×2×3	–347393.8068
Cu_3PO_4	ICSD 427086 [22]	P-3 (147)	5×4×4	–1116292.631
LiCN	ICSD 77321 [23]	Pnma (62)	2×4×2	–79922.53618
CuCN	MP 35308	R-3m (166)	3×3×3	–336376.5932
LiSCN	ICSD 425061 [24]	Pnma (62)	1×4×3	–165329.6032
CuSCN	ICSD 32578 [25]	P63mc (186)	4×4×1	–421756.9049
LiH	ICSD 61749 [26]	Fm-3m (225)	5×5×5	–21179.32676
CuH	ICSD 44859 [27]	P6$_3$mc	9×9×6	–277718.1794

Section S3. Synthesis of chemicals

β-LiGaO₂

β-$LiGaO_2$ powder was synthesized by Reaction (S1) from Li_2CO_3 (99%, Fujifilm Wako, Japan) and Ga_2O_3 (99.9%, Kojundo Chemical, Japan): [28]

$$Li_2CO_3\ (s) + Ga_2O_3\ (s) \rightarrow LiGaO_2\ (s) + CO_2\ (g) \qquad (S1)$$

Li_2CO_3 and Ga_2O_3 were weighed in a ratio of Li:Ga = 1.06:1 to consider Li evaporation, then mixed using a planetary ball mill. The mixture was formed into pellets through uniaxial pressing at 100 MPa and sintered at 600 °C for 48 h in the air.

Cu_2SO_4

Cu_2SO_4 was synthesized by Reaction (S2) using fine-particle Cu_2O (FRC-05B, Furukawa Chemicals, Japan) and $(CH_3)_2SO_4$ (dimethyl sulfate, Fujifilm Wako Pure Chemicals, Japan).[19] The Cu_2O used consisted of extremely fine particles, consistent with previous reports that fine-particle Cu_2O is desirable for this synthesis method. [19]

$$Cu_2O\ (s) + (CH_3)_2SO_4\ (\ell) \rightarrow Cu_2SO_4\ (s) + (CH_3)_2O\ (g) \qquad (S2)$$

A 10 mL of $(CH_3)_2SO_4$ and 0.1 g of Cu_2O were combined in a round-bottom flask and stirred at 250 rpm under an Ar atmosphere. The flask was immersed in a 160 °C oil bath and heated for 10 minutes while stirring. Subsequently, the flask was taken out from the oil bath and cooled rapidly in room-temperature water. The supernatant of liquid was decanted, and the precipitate was dried under a vacuum at room temperature.

The acquired Cu_2SO_4 was verified as a single phase in the XRD profile (Figure S1). This Cu_2SO_4 can be preserved in vacuum desiccator or under an inert atmosphere at room temperature for a minimum of several weeks without deterioration.

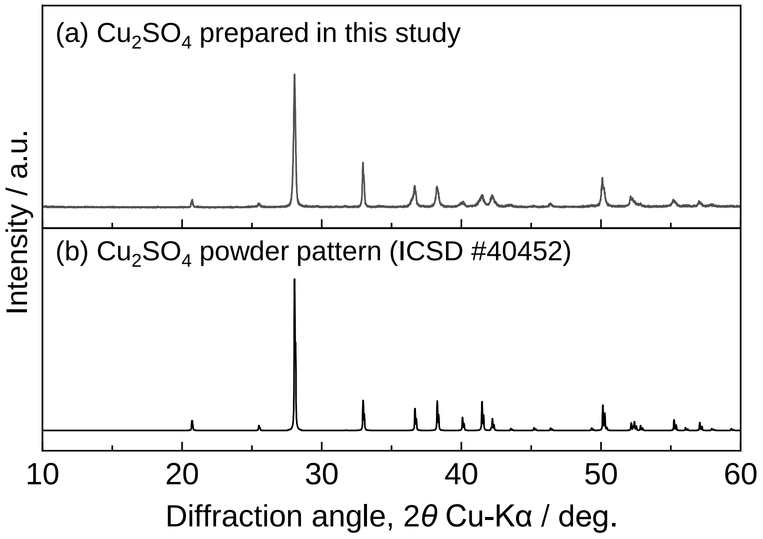

Figure S1. XRD profiles of the obtained Cu_2SO_4 compared with the simulated powder pattern of Cu_2SO_4 (ICSD 40452 [19]).

Cu_3PO_4

Cu_3PO_4 was synthesized via a two-step Reactions (S3, S4).[22]

$$2(NH_4)2HPO_4\ (s) + 3CuO\ (s) \rightarrow Cu_3(PO_4)_2\ (s) + 4NH_3\ (g) + 3H_2O\ (g) \quad (S3)$$

$$Cu_3(PO_4)_2\ (s) + 3Cu\ (s) \rightarrow 2Cu_3PO_4 \quad (S4)$$

($(NH_4)_2HPO_4$ (99.0+%, Fujifilm Wako Pure Chemical) and CuO (99.9%, Fujifilm Wako Pure Chemical) were weighed in a 2:3 molar ratio and mixed with air using a mortar and pestle for 30 minutes. The mixture was then uniaxially pressed at 100 MPa to form Φ9 mm pellets, which were sintered in air at 1000 °C for 40 h to obtain single-phase $Cu_3(PO_4)_2$ (Figure S2(a)). The resulting $Cu_3(PO_4)_2$ was then mixed with metallic Cu powder (99%, ~75 μm, Fujifilm Wako Pure Chemicals) in a 1:3 molar ratio using a mortar and pestle in a glove box filled with a N_2 atmosphere for about 30 min. This mixture was then pressed into Φ 9 mm pellets under 100 MPa and vacuum-sealed in a quartz tube. The tube was heated at 850 °C for 72 h and then rapidly quenched by placing it in water. This process yielded nearly single-phase Cu_3PO_4 (Figure S2(c)). This Cu_3PO_4 can be preserved in vacuum desiccator or under an inert atmosphere at room temperature for a minimum of several months without deterioration.

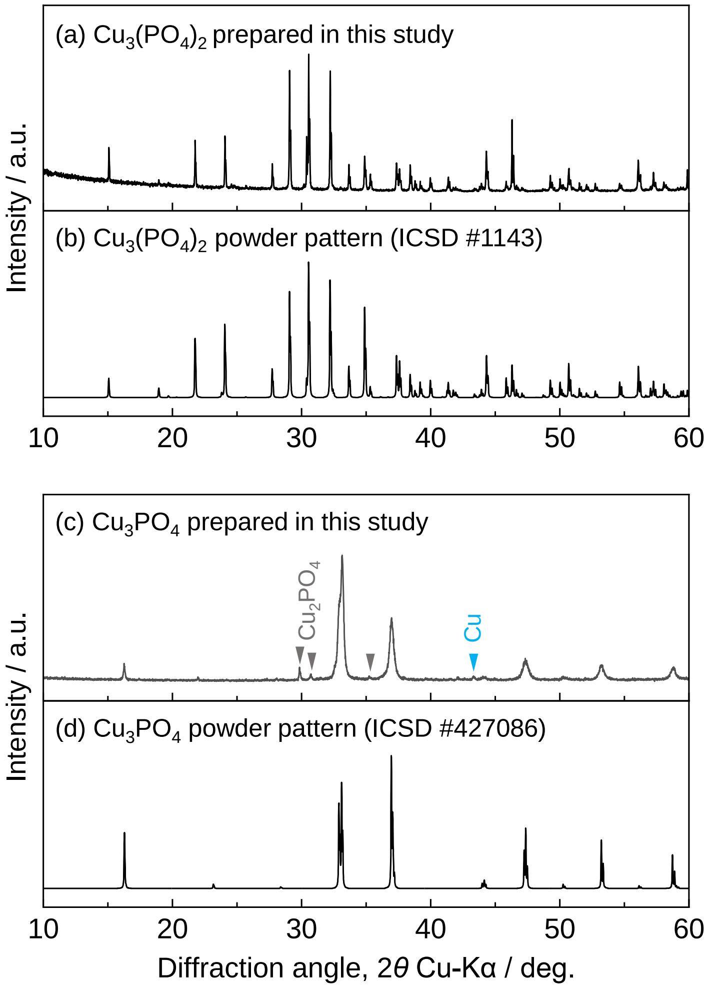

Figure S2. XRD profiles of (a) $Cu_3(PO_4)_2$ and (c) Cu_3PO_4 synthesized in this study, along with the simulated powder patterns of $Cu_3(PO_4)_2$ (ICSD #1143 [29]) and Cu_3PO_4 (ICSD #427086 [22]).

Section S4. Ion-exchange processes

β-LiGaO$_2$ and Cu$_2$SO$_4$ or Cu$_3$PO$_4$

β-LiGaO$_2$ powder and Cu$_2$SO$_4$ or Cu$_3$PO$_4$ powder were mixed in a ratio of Li:Cu = 1:1 in a glove box filled with N$_2$ gas using a mortar and pestle for 15 min. Subsequently, the homogenized mixture was pressed into a Φ9 mm pellet at 100 MPa using a uniaxial press. The pellet was positioned at the base of a Pyrex test tube and subjected to heating at either 150 or 250 °C for 20 h in an electric furnace, while the opposite end of the test tube was connected to a rotary pump. Following the reaction, the sample was rinsed with ultrapure water, and the resulting precipitate was harvested via centrifugation. This washing procedure was iterated thrice, culminating in a final rinse with ethanol. The precipitate, post-final centrifugation, was subsequently dried in a vacuum desiccator.

LiCl and Cu$_3$PO$_4$

LiCl powder (99.9%, Fujifilm Wako) was blended with Cu$_3$PO$_4$ in a ratio of Li:Cu = 1:1 and underwent an ion exchange process similar to the method employed for β-LiGaO$_2$ above. Heating temperature and duration were 150 °C and 20 h, respectively.

Section S5. Rietveld analysis

The ion-exchanged β-$CuGaO_2$, synthesized from β-$LiGaO_2$ and Cu_2SO_4 and subsequently washed, was analyzed by Rietveld refinement using SmartLab Studio II (v4.1.0.191, Rigaku Corp., Japan). The refinement was performed with β-$CuGaO_2$ and Cu_2O as initial input phases, optimizing the lattice parameters and background while excluding atomic coordinates and site occupancies from the refinement process. A split pseudo-Voigt function was used for peak shape modeling, and a B-spline was applied for the background model.

Figure S3 presents the observed diffraction profile, the calculated profile based on the Rietveld refinement, their difference, and the associated fitting indices, demonstrating that the fitting was practically sufficient. Table S3 summarizes the refined lattice parameters of β-$CuGaO_2$ and Cu_2O, the differences from the reported values in the literature, and their molar ratio in the mixture, which corresponds to a Cu:Ga atomic ratio of 1.30:1.

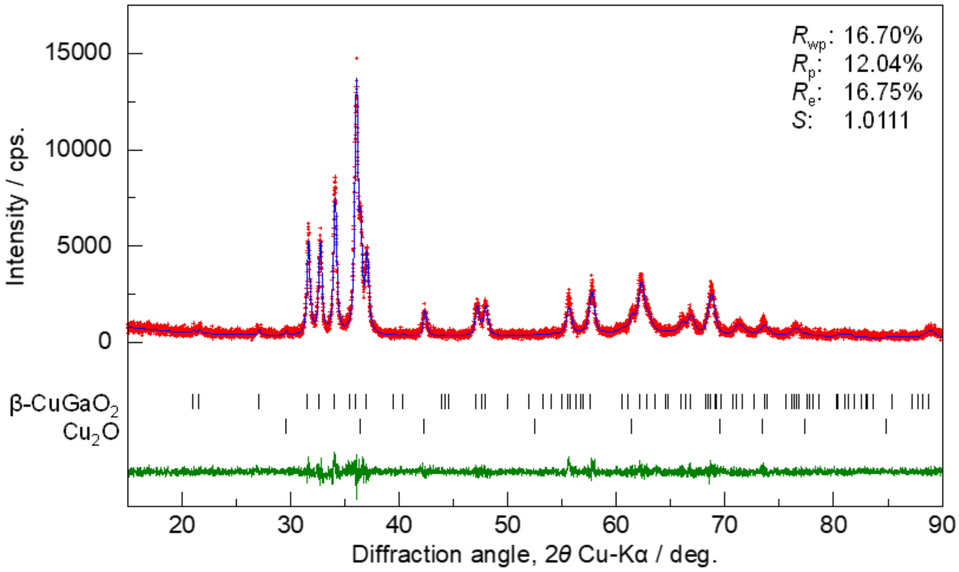

Figure S3. Rietveld refinement plot showing the observed (red crosses), calculated (blue line) and difference (green line) profiles of β-$CuGaO_2$ and Cu_2O, Black vertical tick marks show calculated peak positions of β-$CuGaO_2$ and Cu_2O.

Table S3. Lattice parameters and molar ratios of β-$CuGaO_2$ and Cu_2O determined by Rietveld refinement. Values in parentheses indicate the differences relative to the reported values in the literature (ICSD#291233[6] and ICSD#38233[30] for β-$CuGaO_2$ and Cu_2O, respectively).

	β-$CuGaO_2$	Cu_2O
a_0 / Å	5.473 (+0.24%)	4.273 (+0.35%)
b_0 / Å	6.607 (-0.05%)	—
c_0 / Å	5.260 (-0.27%)	—
Molar ratio	86.8 mol%	13.2 mol%

Section S6. Reaction of β-$LiGaO_2$ and Cu_3PO_4

When Cu_3PO_4 was utilized as the ion source, no reaction occurred upon heating to 200 °C, except for the formation of Cu and Cu_2PO_4 due to partial disproportionation of the ion source (Figure S4(c)). Increasing the reaction temperature to 250 °C led to complete disproportionation of Cu_3PO_4, while β-$LiGaO_2$ remained unaffected (Figure S4(d)).

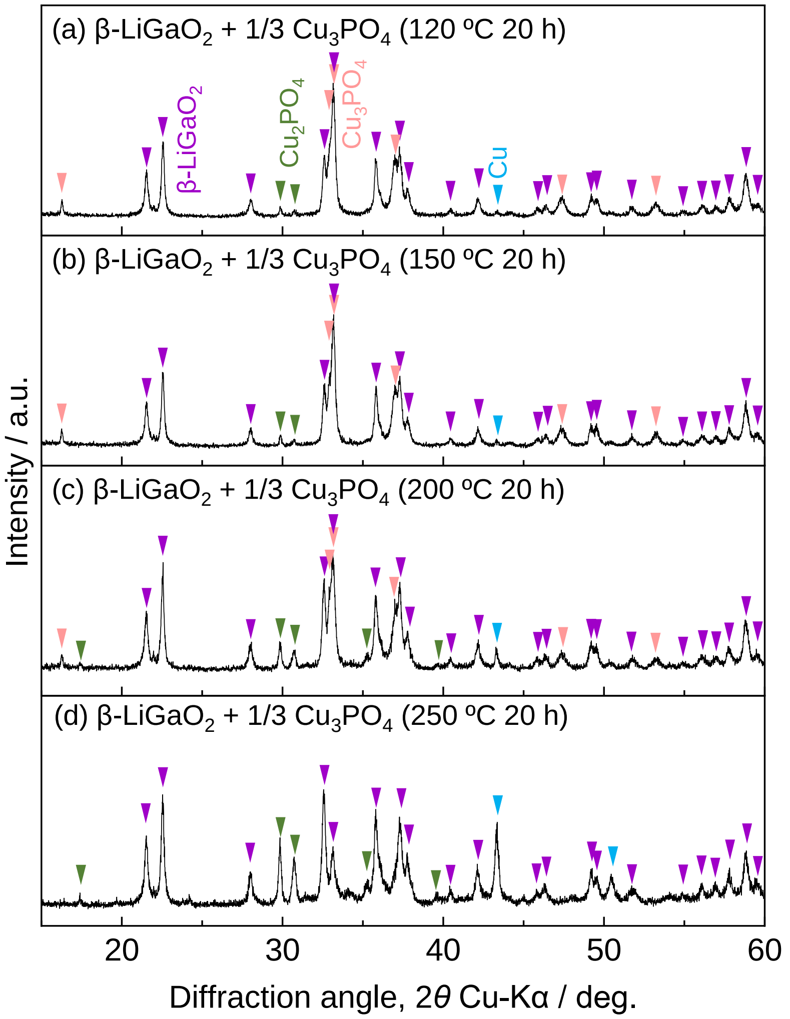

Figure S4. XRD profiles of the samples after the heating process: β-$LiGaO_2$ and Cu_3PO_4 at (a) 120 °C, (b) 150 °C, (c) 200 °C, and (d) 250 °C.

Section S7. Reaction of β-$CuGaO_2$ and Li_3PO_4

As the reverse reaction of Reaction (5) in the main text, Reaction (S5) was conducted. β-$CuGaO_2$ was synthesized through ion exchange between β-$NaGaO_2$ and CuCl, as previously documented.[6] β-$CuGaO_2$ and commercially available Li_3PO_4 (95%, Fujifilm Wako, Japan) were weighed in a ratio of Cu:Li = 1:1, mixed, and then compacted into a pellet. The pellet was heated under vacuum at 250 or 350 °C for 20 h using the same setup as detailed for the ion exchange involving β-$LiGaO_2$ in Section S4.

$$\beta\text{-}CuGaO_2 + \frac{1}{3}Li_3PO_4 \rightarrow \beta\text{-}LiGaO_2 + \frac{1}{3}Cu_3PO_4 \quad \Delta_r H = +6.8\ \text{kJ}\cdot\text{mol}^{-1} \qquad \text{(S5)}$$

As shown in Figure S5, while heating β-$CuGaO_2$ and Li_3PO_4 at 250 °C did not result in any change to β-$CuGaO_2$, heating at 350 °C yields XRD peak shifts. Since the Vegard's law holds for the lattice parameters of the solid solution of β-$CuGaO_2$ and β-$LiGaO_2$,[31] the composition evaluated of the sample from lattice constants was x =0.12 ($Li_{0.12}Cu_{0.88}GaO_2$), as shown in Figure S6. The formation of this solid solution indicates that the increase in entropy gain ($-T\Delta S$) with increasing temperature acted as the primary driving force for ion exchange, supporting the assumption that the $\Delta_r H$ of Reactions (5) and (S5) are almost zero.

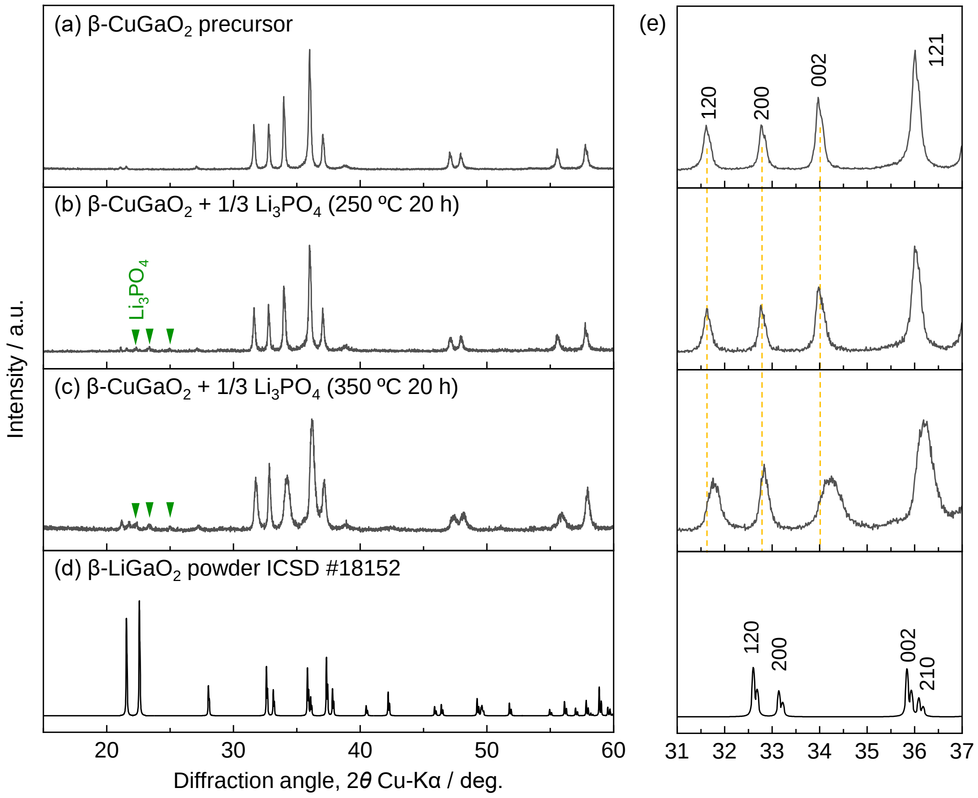

Figure S5. XRD profiles of the sample after the heating process of β-$CuGaO_2$ with Li_3PO_4 (b) at 250 °C and (c) at 350 °C for 20 h, along with the patterns of (a) β-$CuGaO_2$ precursor and (d) β-$LiGaO_2$ powder (ICSD #18152 [8]). (e) Enlarged profiles from 31 to 37°.

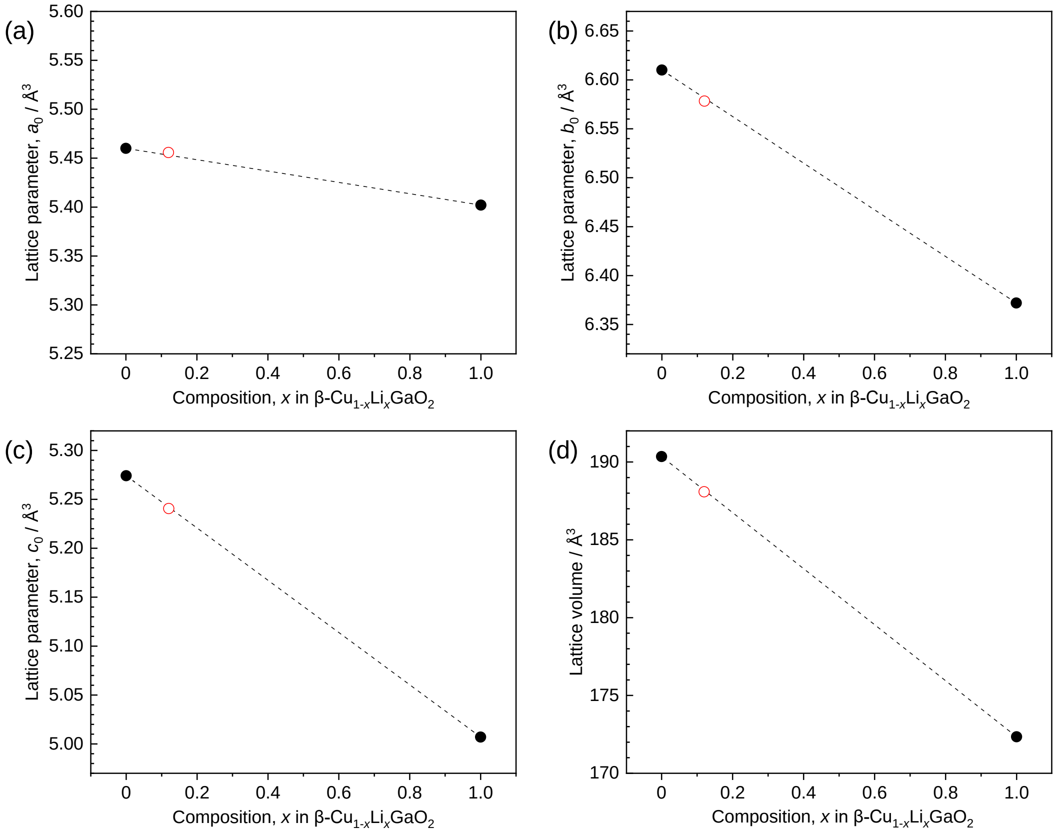

Figure S6. Compositional dependence of (a–c) lattice parameters and (d) lattice volume. The terminals are literature values of β-$CuGaO_2$[6] and β-$LiGaO_2$[8]. The composition of the sample after heating at 350 °C (red open circle figures) corresponds to x = 0.12 ($Li_{0.12}Cu_{0.88}GaO_2$).

References for supporting information

[1] S. Kim, X. Ma, S.P. Ong, G. Ceder, A comparison of destabilization mechanisms of the layered Na_xMO_2 and Li_xMO_2 compounds upon alkali de-intercalation, Phys Chem Chem Phys, 14 (2012) 15571-15578.

[2] R.E. Honig, Vapor pressure data for the solid and liquid elements, RCA review, 30 (1969) 285-305.

[3] H. Ohta, S.-W. Kim, S. Ohta, K. Koumoto, M. Hirano, H. Hosono, Reactive Solid-Phase Epitaxial Growth of Na_xCoO_2 (x~0.83) via Lateral Diffusion of Na into a Cobalt Oxide Epitaxial Layer, Crystal Growth & Design, 5 (2004) 25-28.

[4] Y. Takahashi, K. Kataoka, K.-i. Ohshima, N. Kijima, J. Awaka, K. Kawaguchi, J. Akimoto, Single-crystal synthesis, structure analysis, and physical properties of the calcium ferrite-type $Na_xTi_2O_4$ with $0.558<x<1$, Journal of Solid State Chemistry, 180 (2007) 1020-1027.

[5] S. Suzuki, I. Suzuki, T. Omata, Pulsed laser deposition of β-NaGaO2: significant dependence of sodium fraction, morphology, and phases of the film on deposition position in the plume, Japanese Journal of Applied Physics, 62 (2023).

[6] H. Nagatani, I. Suzuki, M. Kita, M. Tanaka, Y. Katsuya, O. Sakata, S. Miyoshi, S. Yamaguchi, T. Omata, Structural and thermal properties of ternary narrow-gap oxide semiconductor; wurtzite-derived β-$CuGaO_2$, Inorg Chem, 54 (2015) 1698-1704.

[7] H.P. Mueller, R. Hoppe, Zur Kristallstruktur von $KGaO_2$ und $NaGaO_2$(II) [1], Zeitschrift fr anorganische und allgemeine Chemie, 611 (1992) 73-80.

[8] M. Marezio, The crystal structure of $LiGaO_2$, Acta Crystallographica, 18 (1965) 481-484.

[9] P. Giannozzi, S. Baroni, N. Bonini, M. Calandra, R. Car, C. Cavazzoni, D. Ceresoli, G.L. Chiarotti, M. Cococcioni, I. Dabo, A. Dal Corso, S. de Gironcoli, S. Fabris, G. Fratesi, R. Gebauer, U. Gerstmann, C. Gougoussis, A. Kokalj, M. Lazzeri, L. Martin-Samos, N. Marzari, F. Mauri, R. Mazzarello, S. Paolini, A. Pasquarello, L. Paulatto, C. Sbraccia, S. Scandolo, G. Sclauzero, A.P. Seitsonen, A. Smogunov, P. Umari, R.M. Wentzcovitch, QUANTUM ESPRESSO: a modular and open-source software project for quantum simulations of materials, J Phys Condens Matter, 21 (2009) 395502.

[10] P. Giannozzi, O. Andreussi, T. Brumme, O. Bunau, M. Buongiorno Nardelli, M. Calandra, R. Car, C. Cavazzoni, D. Ceresoli, M. Cococcioni, N. Colonna, I. Carnimeo, A. Dal Corso, S. de Gironcoli, P. Delugas, R.A. DiStasio, Jr., A. Ferretti, A. Floris, G. Fratesi, G. Fugallo, R. Gebauer, U. Gerstmann, F. Giustino, T. Gorni, J. Jia, M. Kawamura, H.Y. Ko, A. Kokalj, E. Kucukbenli, M. Lazzeri, M. Marsili, N. Marzari, F. Mauri, N.L. Nguyen, H.V. Nguyen, A. Otero-de-la-Roza, L. Paulatto, S. Ponce, D. Rocca, R. Sabatini, B. Santra, M. Schlipf, A.P. Seitsonen, A. Smogunov, I. Timrov, T. Thonhauser, P. Umari, N. Vast, X. Wu, S. Baroni, Advanced capabilities for materials modelling with Quantum ESPRESSO, J Phys Condens Matter, 29 (2017) 465901.

[11] A. Jain, S.P. Ong, G. Hautier, W. Chen, W.D. Richards, S. Dacek, S. Cholia, D. Gunter, D. Skinner, G. Ceder, K.A. Persson, Commentary: The Materials Project: A materials genome approach to accelerating materials innovation, APL Materials, 1 (2013).

[12] H. Ott, Die Raumgitter der Lithiumhalogenide, Physikalische Zeitschrift, 24 (1923) 209-213.
[13] S. Hull, D.A. Keen, High-pressure polymorphism of the copper(I) halides: A neutron-diffraction study to ~10 GPa, Phys Rev B Condens Matter, 50 (1994) 5868-5885.
[14] G.I. Finch, S. Fordham, The effect of crystal-size on lattice-dimensions, Proceedings of the Physical Society, 48 (1936) 85-94.
[15] D. Fischer, A. Müller, M. Jansen, Existiert eine Wurtzit-Modifikation von Lithiumbromid? – Untersuchungen im System LiBr/LiI–, Zeitschrift für anorganische und allgemeine Chemie, 630 (2004) 2697-2700.
[16] R. Kurdyumova, R. Baranova, An electron diffraction study of thin films of cuprous iodide, Kristallografiya, 6 (1961) 402-405.
[17] N.W. Alcock, D.A. Evans, H.D.B. Jenkins, Lithium sulphate – a redetermination, Acta Crystallographica Section B Structural Crystallography and Crystal Chemistry, 29 (1973) 360-361.
[18] D.C. Parfitt, D.A. Keen, S. Hull, W.A. Crichton, M. Mezouar, M. Wilson, P.A. Madden, High-pressure forms of lithium sulphate: Structural determination and computer simulation, Physical Review B, 72 (2005).
[19] H.J. Berthold, J. Born, R. Wartchow, The crystal structure of copper(I)sulfate Cu_2SO_4, Zeitschrift für Kristallographie - Crystalline Materials, 183 (1988) 309-318.
[20] C. Keffer, A.D. Mighell, F. Mauer, H.E. Swanson, S. Block, Crystal structure of twinned low-temperature lithium phosphate, Inorganic Chemistry, 6 (2002) 119-125.
[21] O. Yakubovich, V. Urusov, Electron density distribution in lithiophosphatite Li_3PO_4. Crystallochemical features of orthophosphate groups with hexagonal close packing, Kristallografiya, 42 (1997) 301-308.
[22] K. Snyder, B. Raguž, W. Hoffbauer, R. Glaum, H. Ehrenberg, M. Herklotz, Lithium Copper(I) Orthophosphates $Li_{3-x}Cu_xPO_4$: Synthesis, Crystal Structures, and Electrochemical Properties, Zeitschrift für anorganische und allgemeine Chemie, 640 (2014) 944-951.
[23] J.A. Lely, J.M. Bijvoet, The crystal structure of lithium cyanide, Recueil des Travaux Chimiques des Pays-Bas, 61 (1942) 244-252.
[24] O. Reckeweg, A. Schulz, B. Blaschkowski, T. Schleid, F.J. DiSalvo, Single-crystal structures and vibrational spectra of Li[SCN] and Li[SCN]·$2H_2O$, Zeitschrift für Naturforschung B, 69 (2014) 17-24.
[25] D.L. Smith, V.I. Saunders, Preparation and structure refinement of the 2H polytype of β-copper(I) thiocyanate, Acta Crystallographica Section B Structural Crystallography and Crystal Chemistry, 38 (1982) 907-909.
[26] J.P. Vidal, G. Vidal-Valat, Accurate Debye–Waller factors of 7LiH and 7LiD by neutron diffraction at three temperatures, Acta Crystallographica Section B Structural Science, 42 (1986) 131-137.
[27] J.A. Goedkoop, A.F. Andresen, The crystal structure of copper hydride, Acta Crystallographica, 8 (1955) 118-119.
[28] T. Omata, K. Tanaka, A. Tazuke, K. Nose, S. Otsuka-Yao-Matsuo, Novel wide band gap alloyed

semiconductors, $x(LiGaO_2)_{1/2}$-$(1-x)$ZnO, and fabrication of their thin films, Science in China Series E: Technological Sciences, 52 (2009) 111-115.

[29] G.L. Shoemaker, J.B. Anderson, E. Kostiner, Copper(II) phosphate, Acta Crystallographica Section B Structural Crystallography and Crystal Chemistry, 33 (1977) 2969-2972.

[30] T. Yamaguti, An Investigation on Oxidation of Crystal Surfaces with Electron Diffraction Method, II. Copper Single Crystals, Proceedings of the Physico-Mathematical Society of Japan. 3rd Series, 20 (1938) 230-241.

[31] I. Suzuki, Y. Mizuno, T. Omata, Tunable Direct Band Gap of β-$CuGaO_2$ and β-$LiGaO_2$ Solid Solutions in the Full Visible Range, Inorg Chem, 58 (2019) 4262-4267.
